# Examining Plausibility of Self-Reported Energy Intake Data: Considerations for Method Selection

**DOI:** 10.3389/fnut.2017.00045

**Published:** 2017-09-25

**Authors:** Jinan C. Banna, Megan A. McCrory, Marie Kainoa Fialkowski, Carol Boushey

**Affiliations:** ^1^University of Hawaii at Manoa, Honolulu, HI, United States; ^2^Boston University, Boston, MA, United States; ^3^University of Hawaii Cancer Center, Honolulu, HI, United States

**Keywords:** misreporting, plausibility, reported energy intake, Goldberg cutoff, estimated energy requirement

## Abstract

Self-reported dietary intake data contain valuable information and have long been used in the development of nutrition programs and policy. Some degree of measurement error is always present in such data. Biological plausibility, assessed by determining whether self-reported energy intake (rEI) reflects physiological status and physical activity level, must be examined and accounted for before drawing conclusions about intake. Methods that may be used to account for plausibility of rEI include crude methods such as excluding participants reporting EIs at the extremes of a range of intake and individualized methods such as statistical adjustment and applying cutoffs that account for the errors associated with within-participant variation in EI and total energy expenditure (TEE). These approaches allow researchers to determine how accounting for under- and overreporting affects study results and to appropriately address misreporting in drawing conclusions with data collected and in interpreting reported research. In selecting a procedure to assess and account for plausibility of intake, there are a number of key considerations, such as resources available, the dietary-report instrument, as well as the advantages and disadvantages of each method. While additional studies are warranted to recommend one procedure as superior to another, researchers should apply one of the available methods to address the issue of implausible rEI. If no method is applied, then at minimum, mean TEE or rEI/TEE should be reported to allow readers to ascertain the degree of misreporting at a gross level and better interpret the data and results provided.

## Introduction

Self-reported dietary intake data contain valuable information and have long been used in the development of nutrition programs and policy. Some degree of measurement error is always present in such data (1). In a recent article, Subar et al. point to the importance of acknowledging measurement error and developing methods to mitigate error in self-report intake data (2). The authors provide the following recommendation: acknowledge the limitations and analyze and interpret self-report dietary data appropriately. Biological plausibility, assessed by determining whether self-reported energy intake (rEI) reflects physiological status and physical activity level (PAL), must be examined and accounted for before drawing conclusions about intake. Key terms related to plausibility of rEI data are presented in Table 1. In this study, the primary objectives are to: (1) provide a guide to key terms that will allow readers to better interpret study results related to assessment of plausibility of rEI data; (2) briefly introduce methods that may be used to account for plausibility of rEI, and (3) present and discuss studies that have compared different methods used to account for plausibility of rEI data.

**Table 1:** Definition of key words related to topic of plausibility of self-report energy intake data.

Key word/phrase	Definition
Biological/physiological plausibility	Indicates whether rEI reflects physiological status and PAL. To improve the validity of self-reported energy data, biological plausibility may be accounted for during analysis (3)
Biologically/physiologically implausible reporting	Refers to rEI that is outside the expected range given physiological status and PAL, or misreporting of energy intake. Such misreporting produces error in dietary assessment. Misreporting includes both over- and underreporting and affects the validity of the data collected and conclusions drawn. Underreporting is a more common problem than overreporting (4)
rEI	Indicates the energy (kcal) consumed in a defined period using a self-report measure of intake such as 24-h dietary recall or dietary record. To determine biological plausibility, rEI is compared with TEE to identify implausible reports that may be screened out using cutoffs (5)
TEE	Refers to energy expenditure in a defined period. TEE may be either measured with techniques such as doubly labeled water, predicted by EER equations, or determined by multiplying BMR or RMR by an activity factor, where the activity factor is either assumed or using methods such as a self-report questionnaire (5)
pER	The mean daily energy intake predicted to maintain energy balance in a healthy adult (5)
REE	The energy expended by a person at rest, accounting for the majority of 24-h energy expenditure. REE is considered to be synonymous with RMR and is used along with PAL to determine pER. REE can be either measured or predicted using prediction equations (5)
BMR	The energy needed to sustain the metabolic activities of cells and tissues, plus the energy needed to maintain blood circulation, respiration, and gastrointestinal and renal function while awake, in a fasting state, and resting comfortably in a thermoneutral environment (5)
PAL	Commonly described as the ratio of total to basal daily energy expenditure. PAL is used in the calculation of TEE and EER (5)
EER	EER prediction equations for normal-weight men and women, overweight men and women, normal weight/overweight men and women combined, and pregnant women, lactating women, and children have been derived from a database of over 1,000 people with DLW measurements of TEE. Two prediction equations for men are presented below (5) TEE (normal and overweight/obese men ages 19 years and older) is calculated with the following formula: TEE = 864 − (9.72 × age [years]) + PA × (14.2 × weight [kg] + 503 × height [m]) EER (normal-weight men ages 19 years and older) is calculated using the following formula: EER = 662 − (9.53 × age [years]) + PA × (15.91 × weight [kg] + 539.6 × height [m])

*rEI, reported energy intake; TEE, total energy expenditure; EER, estimated energy requirement; BMR, basal metabolic rate; RMR, resting metabolic rate; pER, predicted energy requirement; REE, resting energy expenditure; PAL, physical activity level; DLW, doubly labeled water; PA, physical activity*.

## Accounting for Plausibility of rEI

In any study involving analysis or interpretation of rEI data, researchers should apply a method to address the issue of implausible rEI. The different methods currently available are presented below. Regardless of the method used, the first step is to select an assessment method for energy requirement. Energy expenditure determined by the doubly labeled water (DLW) method is widely accepted as a gold standard for energy requirement to which rEI data may be compared (6). Energy expenditure serves as a biomarker of energy intake when body weight is relatively stable (7) or when there is weight change and the energy cost of weight change is estimated (8). Validation studies have shown that the DLW technique has an accuracy of 1–3% and a precision of 2–8%, when the method is compared with respirometry (9). While DLW is preferred over respirometry given the difficulty of use of respirometry in community dwelling conditions (6), the use of DLW is often not practical given the high costs of isotopes and equipment for isotope analysis, as well as the expertise required for analysis (6). Energy requirement may therefore be predicted by other methods, such as estimated energy requirement (EER) equations, or by multiplying basal metabolic rate (BMR) or resting metabolic rate by an activity factor, where the activity factor is either assumed or assessed by using a method such as a validated questionnaire or an objective method such as a validated accelerometer (10).

## Specific Methods to Account for rEI Plausibility

Crude methods of accounting for plausibility such as excluding participants reporting EIs at the extremes of a range of intake and individualized methods such as statistical adjustment and applying cutoffs that account for the errors associated with within-participant variation in EI and total energy expenditure (TEE) will be briefly presented. A full description of each method is beyond the scope of this study, and readers should refer to the original references to learn more about each method. The different approaches described in this study are summarized in Table 2.

**Table 2:** Overview of methods used to account for plausibility of rEI.

Methods used to account for plausibility of rEI	Description of approach	Strengths	Limitations
Excluding participants who report EIs at the low and high end of a range from the analysis	A commonly used method is to exclude participants who report consuming fewer than 500 and greater than 3,500 cal per day	Provides a consistent protocol when the dietary-report instrument does not allow use of the computational energy cutoff methods	Crude method that is not individualized May not identify all implausible reports of EI
Goldberg CUT-OFF 2 (11)	Based on number of days of self-report, coefficients of variation for EI, estimated BMR, PAL, and sample size	Individualized method of assessing plausibility of rEI	Error in assigning PAL is not accounted for Only identifies extremely inaccurate reporting
Method introduced by McCrory et al. (12) and updated by Huang et al. (3)	Cutoffs for rEI are calculated as a percentage of pER specific to sex and age per the DRI categories and weight status	Takes into account the within-subject errors in TEE and rEI, including measurement error and normal day to day variation Simple and individualized approach to assessing plausibility of rEI	Using Huang et al.’s updated method (3), the error in assigning PAL if calculating EER is not considered
Calculation of the ratio of rEI:pER and statistical adjustment using this value	rEI:pER included as a confounding factor in a statistical model	Sample size remains intact Can reduce measurement error because errors in intakes tend to be highly correlated and partly cancel each other with adjustment for energy intake	Assumes that the macronutrients are underreported proportionately

*rEI, reported energy intake; EI, energy intake; BMR, basal metabolic rate; PAL, physical activity level; pER, predicted energy requirement; DRI, dietary reference intake; TEE, total energy expenditure; EER, estimated energy requirement*.

### Exclusion of Participants with High or Low rEI (Non-Individualized Method)

A crude method to account for plausibility of rEI involves excluding from the analysis participants reporting EIs at the extreme low and high ends of rEI (13). While the specific values used differ among studies, common values used to exclude participants include fewer than an average of 500 and greater than 3,500 kcal per day over time for women (14–19) and fewer than an average of 800 and greater than 4,200 kcal per day for men (14, 19). Willett notes that the range of 500–3,500 kcal/day may be applied to data from women, and an allowable range of 800–4,000 kcal/day for men may be used, as intakes of more than 4,000 kcal/day are unlikely to be true for even relatively active men (13). An advantage of this method is that it provides a consistent protocol when the dietary-report instrument does not allow for an accurate estimation of energy intake, as is the case with food frequency questionnaires (FFQs), whether they focus on intake of specific nutrients or foods (20, 21) or attempt to comprehensively assess diet. However, a drawback of this crude approach is that it is not individualized and does not capture all implausible reports. This is illustrated by observed TEE values greater than 3,500 kcal/day in obese and extremely obese men and women with low or normal PALs, and in normal weight, very active individuals (22–24). Therefore, using a blanket assumption that all rEIs exceeding a given value are implausible may be inappropriate depending on the characteristics of participants within a dataset.

### Use of Cutoffs for Under- and Overreporting of rEI (Individualized Method)

When assessing plausibility of rEI, cutoffs for rEI may be used to distinguish and group misreporters, or implausible reporters (i.e., under- or overreporters), separately from acceptable reporters, or plausible reporters. Goldberg et al. derived one such cutoff limit, known as CUT-OFF 2 (11). This method uses the number of days of self-reported intake, sample size and within-day coefficients of variation for rEI, estimated BMR, and PAL to determine low and high cutoff values [e.g., 95% confidence limits (CL)], as follows:

$$\text{Lower and upper }95\text{\% CL}=\text{square root of }(\text{C}{\text{V}}_{\text{wEI}}^{2}/d+\text{C}{\text{V}}_{\text{wB}}^{2}+\text{C}{\text{V}}_{\text{tP}}^{2}),$$

where CV_wEI_ is the within-subject coefficient of variation in EI across days, *d* is the number of days of diet assessment, CV_wB_ is the coefficient of variation of repeated BMR measurements of the precision of estimated compared with measured BMR, and CV_tP_ is the total variation in PAL and takes into account both between and within-subject variation as well as methodological errors in PAL. We refer the reader to the original reference (11) for a full explanation of the use of this equation.

Strengths of the method include accounting for the within-subject errors in TEE and rEI, including measurement error and normal day to day variation. This method represents a simple individualized approach to assessing plausibility of rEI. Several limitations of this approach have also been noted, such as the failure to account for the error in assigning PAL and the lack of ability to identify misreporting that is only mildly inaccurate (12). In a study seeking to quantify the accuracy of the Goldberg method for categorizing misreporters, Tooze et al. (25) demonstrated that the Goldberg method was able to adequately discriminate between underreporters and acceptable reporters. For these sensitivity and specificity analyses, the authors excluded overreporters due to the small number of participants classified as such.

An additional method was introduced by McCrory et al. (12) and updated in a subsequent paper by the same group (3). Like the Goldberg method, this method takes into account the within-subject errors in TEE and rEI, including measurement error and normal day to day variation, and is a simple and individualized approach to assessing plausibility of rEI (3). Using this method, 1 SD cutoffs for rEI are calculated as a percentage of predicted energy requirement (pER) specific to sex and age and weight status as follows:

$$\pm 1\text{SD}=\text{square root of }(\text{C}{\text{V}}_{\text{rEI}}^{2}/d+\text{C}{\text{V}}_{\text{pER}}^{2}+\text{C}{\text{V}}_{\text{mTEE}}^{2}),$$

where CV_rEI_ is the within-subject coefficient of variation in rEI across days, *d* is the number of days of intake, CV_pER_ is the coefficient of variation of pER (taken as the root mean squared error of the prediction equation), and CV_mTEE_ is the within-person CV of measured TEE and takes into account the measurement error and day to day biological variation in TEE [taken as 8.2%, from Black et al. (26)]. We refer the reader to the original reference (3) for a full explanation of the use of this equation.

However, the method could be adapted to use TEE measured by DLW, predicted by any prediction equation (Table 1), or estimated using a questionnaire such as the Minnesota Leisure Time Physical Activity Questionnaire (27). A limitation of the method, as with Goldberg CUT-OFF 2, is that the error in assigning PAL during calculation of EER, if EER is used as the method to estimate energy requirement, is not considered.

### Statistical Adjustment Using rEI:TEE or rEI:pER (Individualized Method)

Conclusions put forth by Tooze et al. (25) were that the use of cutoffs to exclude implausible reporters may lead to loss of statistical power and biased estimates of associations of both underreporting with personal characteristics and dietary variables with outcomes. Another problem in applying cutoffs is that body weight is included in both the calculation of implausible rEI and the outcome variable in subsequent analyses of how dietary variables are associated with the outcome (13), which could artificially elevate the association. These drawbacks must be considered when applying either of these cutoff methods. One approach that has been used that avoids these potential pitfalls is to calculate the ratio of rEI:TEE or rEI:pER and include one of these ratios in regression models of dietary variables predicting health outcomes such as risk for overweight or obesity, thereby statistically adjusting for energy intake misreporting. Murakami and Livingstone recently used this method in a study examining associations of eating frequency, meal frequency, and snack frequency with adiposity and found positive associations between eating frequency and overweight/obesity that were inverse or null before EI:EER was taken into account (28). Mendez et al. also adjusted for misreporting in a study examining associations between dietary factors and BMI and found that adjusting for the ratio versus excluding implausible reporters yielded qualitatively similar results (29). The authors suggest that adjusting is a viable alternative to omitting a substantial proportion of the study sample. In examining misreporting, however, other studies have shown that individuals tend to underreport macronutrients disproportionately, with carbohydrates, fat, and alcohol underreported to a greater degree than protein (30–32). This does pose a problem when the assumption underlying the adjustment for misreporting is that macronutrients are reported proportionately.

## Research Comparing Different Methods to Account for Plausibility of rEI

A small number of studies have compared different methods used to account for plausibility of rEI data. In a study seeking to elucidate the methods to best identify and account for misreporting, Mendez et al. identified misreporters on the basis of disparities between rEI and TEE (29). The authors performed the calculation using the Goldberg CUT-OFF 2 method and two alternatives: one that substituted BMR equations that are more valid at higher BMIs (11, 33) and the method of Huang et al. described earlier (3, 12). Results indicated that levels of underreporting were lower and levels of overreporting higher using both of the alternative methods compared to results using the Goldberg CUT-OFF 2 method, with the two alternative methods yielding concordant results in their subsequent examination of the relationship between dietary factors and weight status. Mendez et al. also applied the crude cutoff method of excluding participants consuming fewer than 500 and greater than 3,500 kcal per day and found that results differed from when using alternative methods. When these participants were excluded rather than using EERs to identify implausible reporters, coefficients in all models were similar to baseline multivariate models (29).

In another study, Rhee et al. presented a comparison of three methods to account for implausible reporting of intake in epidemiologic studies (34). In addition to excluding participants according to rEI (<500 and >3,500 kcal/day), the authors also used the Goldberg CUT-OFF 2 method and predicted total energy expenditure (pTEE) (3) methods to classify under- and overreporters. All results concerning the relationships between the dietary variables and outcome were qualitatively similar with the application of each method. However, the two latter methods estimated a higher prevalence of under- and overreporters than the first, leading the authors to conclude that using either of the latter individualized methods did not provide a major advantage in detecting diet–BMI associations over the crude method. In the carotenoids and retinol sample, for example, 98.9% of participants were classified as plausible reporters using the crude method, while only 71.1 and 69.6% were classified as such using the Goldberg CUT-OFF 2 and pTEE methods, respectively (34). This study included only women; thus, additional studies in men and children would be needed before applying these results more broadly.

Jessri et al. evaluated several methods for accounting for implausible reporting among a nationally representative sample in Canada as part of an examination of the association between dietary factors and obesity (35). Included in this analysis was a propensity score, which is a statistical technique used to reduce bias by equating groups based on variables associated with misreporting (36, 37). The methods examined were as follows: (1) statistically adjusting for variables related to misreporting; (2) excluding misreported recalls; (3) statistically adjusting for reporting groups (underreporters, plausible reporters, and overreporters); (4) statistically adjusting for propensity score; and (5) stratifying the analyses by reporting groups (35). Results indicated that exclusion of misreporters, adjusting for the reporting groups, and stratification yielded risk estimates that were more consistent than the other methods with expectations regarding the relationship between dietary factors and obesity. There was no benefit of adjusting for the propensity score over adjusting for the reporting group with regard to improving the association between dietary factors and obesity.

## Inappropriate Methods to Account for Plausibility of EI

In addition to the aforementioned procedures that may be used to account for plausibility of EI, there are several other methods that are inappropriate for use, but are still being employed. The first is another cutoff limit derived by Goldberg et al., known as CUT-OFF 1 (11). Using this method, an absolute value of 1.35 for EI:BMR is used, assuming that BMR had been measured rather than predicted and that the rEI represented habitual intake. Black notes that this strategy ignores the errors associated with within-subject variation in EI and TEE and should not be employed in accounting for plausibility of EI (26). The use of a single cutoff for EI:BMR for all participants fails to identify underreporters among those with high energy requirements and is inappropriate for use (26). Further, it does not take overreporting into account. A second inappropriate method is that a value of rEI/pER < 1.0 is classified as underreporting, and a value of rEI/pER > 1.0 is classified as overreporting. When this is done, measurement error and day to day variation in EI and TEE are not taken into account and perfection is assumed. While it is inappropriate to classify participants as under- or overreporters based on these values, researchers may wish to report the number of participants falling above and below rEI/pER = 1.0 to describe the study sample. Researchers may also use this calculation as a covariate as described earlier.

## Conclusion

As misreporting of dietary intake is a well-documented problem across population groups, it is imperative that plausibility of rEI be addressed in studies examining dietary intake. Any of the methods described in this study may be applied to data collected in children, adolescents, adults, and elderly individuals provided an estimate of energy requirement is available. In this study, both crude methods of accounting for plausibility such as excluding participants reporting EIs at the extremes of a range of intake and individualized methods such as adjustment and cutoffs that account for the errors associated with within-participant variation in EI and TEE have been described. These approaches allow researchers to determine how accounting for under- and overreporting affects study results and to appropriately address misreporting in drawing conclusions with data collected and in interpreting reported research.

In selecting a procedure to assess and account for plausibility of intake, there are a number of key considerations, such as resources available, the dietary-report instrument, as well as the advantages and disadvantages of each method. DLW may be used or another technique to assess energy requirement when resources are limited. If the dietary-report instrument is not able to yield an accurate estimation of EI, as is the case with FFQs, then a crude method to account for misreporting may be the best option. However, further study is needed in this area to determine the most appropriate method to account for misreporting when using such dietary-report instruments. Regardless of which method is used, for the time being, analyses in the total sample without exclusion of participants should also be conducted and reported. This will allow the researcher to examine the impact of using each method, and will also address the potential bias that may be introduced when participants are excluded from the analysis. While additional studies are warranted to recommend one procedure as superior to another, researchers should apply one of the available methods to address the issue of implausible rEI. If no method is applied, then at minimum, mean TEE or rEI/TEE should be reported to allow readers to ascertain the degree of misreporting at a gross level and better interpret the data and results provided.

## Author Contributions

All authors jointly conceived of the content of the manuscript. JB and MF drafted the manuscript. All the authors participated in critical revision of the manuscript.

## Conflict of Interest Statement

The authors declare that the research was conducted in the absence of any commercial or financial relationships that could be construed as a potential conflict of interest.
